# Challenges with Conducting an Investigational Drug Study in Older Adults in Nursing Homes During a Pandemic

**DOI:** 10.1093/geroni/igab046.2694

**Published:** 2021-12-17

**Authors:** H Edward Davidson, Lisa Han, Melissa LaMantia, Michelle Trout, Joan Mannick, Stefan Gravenstein

**Affiliations:** 1 Insight Therapeutics, LLC, Norfolk, Virginia, United States; 2 Insight Therapeutics, LLC, Insight Therapeutics, LLC, Virginia, United States; 3 Life Biosciences, Boston, Massachusetts, United States; 4 Brown University, Providence, Rhode Island, United States

## Abstract

In the early months of the pandemic, SARS-CoV-2 infected nursing home residents in explosive and deadly outbreaks. Nursing home residents disproportionately accounted for over 40% of COVID-19 mortality nationally. This national emergency drove scientific and public health experts to develop and implement administrative, clinical, and research programs to limit the pandemic’s impact, especially for high-risk individuals, such as those hospitalized or living in nursing homes. Nursing home policies, prompted by the Centers for Medicare & Medicaid Services (CMS) and the Centers for Disease Control and Prevention (CDC) guidance, severely restricted access beginning in March 2020 in an effort to limit disease exposure. In July 2020 we began the process to conduct an investigational SARS-CoV-2 post exposure prophylaxis study of nursing home residents, incorporating FDA guidance developed for conducting investigational drug trials in the context of COVID-19. Our research teams adapted our nursing home engagement, resident consenting and research data collection strategies accordingly. We remotely screened residents living in any of 28 nursing homes for eligibility to participate, ultimately consenting and randomizing individuals in 11 facilities. Of the 2,683 nursing home residents 65 years or older we screened, 48 (1.8%) agreed to consent individually or through proxy, most often a legally authorized representative. We will describe our research methods, with emphasis on how we addressed challenges presented due to performing all research tasks remotely and identify strategies that can qualitatively improve the remote nursing home research experience.

